# Supplementary material for: New circulation of genotype V of Crimean-Congo haemorrhagic fever virus in humans from Spain
Source: PLoS Negl Trop Dis. 2021 Feb 22;15(2):e0009197. doi: 10.1371/journal.pntd.0009197 (PMC7943016; doi:10.1371/journal.pntd.0009197)
Supplement: Attachment — Submitted filename: letterPlosNTD.docx [file pntd.0009197.s001.docx]

**Reviewer's Responses to Questions**

**Reviewer #2:**

A retrospective study of sera was collected from various areas in Spain from folks that had any clinical presentations included in a typical CCHF case definition. These sera were screened using various antigenic and nucleic methods (ELISA/PCR/Sanger etc.,) which revealed four retrospective cases of CCHF. The virus was interestingly typed to the European clade, suggesting that both circulating African clade and European clade viruses were present and infecting both ticks and humans within Spanish borders.

Methods are appropriate for the study aims. This reviewer would recommend to including the type of reagents used in the in-house ELISA and IFA assay used for detection? What were the antigens or antibodies utilized as well as the methodologies?

Nucleoprotein (NP) and glycoprotein (GPC) antigens at 1:10 dilution was used to confirm the positive results.

**Minor comments:**

The positive acute case was associated with cattle husbandry and the virus clusters with Turkey, Kosovo and Russian strains of CCHFV. Is there any movement of cattle between these regions and does the patient have any history of travel to these regions? If not, can authors speculate on how CCHFV from this region spread to Spain? Are there migratory birds that travel between these regions? Livestock trade?

The patient did not travel to Turkey, Kosovo, Russia or any other Eastern European country. Our hypothesis is that this novel variant of the virus was introduced in the Spanish territory by livestock displacement through (legal or illegal) animal trade from zones of East, Centre Europe to Spain. It is known that genotype III was introduced in our territory by migratory birds from Western Africa (10). So far, principal direction of bird migration is north-south, and birds from Eastern countries of Europe fly in autumn through Turkey to reach Africa, however, the possibility of the introduction of the genotype V by migratory birds cannot be completely ruled out. Further studies should be carried out in order to clarify how this novel strain has reach the Spanish territory.

Patient population presented with an undifferentiated febrile illness and only one patient was positive for CCHFV. Do authors have any data on what the rest of the patients had?

Authors do not have the data of these other diagnosis.

Line 288 is missing a reference.

Reference has been included.

Authors state that their qRT-PCR was modified to include an internal control. What was this control?

Internal control consisted in a heterologous DNA fragment of 135 pb flanked by nucleotide primer pair included into reaction mix.
